# The BBIBP-CorV inactivated COVID-19 vaccine induces robust and persistent humoral responses to SARS-CoV-2 nucleocapsid, besides spike protein in healthy adults

**DOI:** 10.3389/fmicb.2022.1008420

**Published:** 2022-11-04

**Authors:** Qinjin Wang, Jie Ning, Ying Chen, Bin Li, Liang Shi, Taojun He, Fang Zhang, Xingchi Chen, Aixia Zhai, Chao Wu

**Affiliations:** Department of Laboratory Medicine, The Eighth Affiliated Hospital of Sun Yat-sen University, Shenzhen, China

**Keywords:** SARS-CoV-2, inactivated vaccine, BBIBP-CorV, nucleocapsid, epitope

## Abstract

Vaccination is one of the best ways to control the severe acute respiratory syndrome coronavirus 2 (SARS-CoV-2) epidemic. Among the various SARS-CoV-2 vaccines approved for use, the BBIBP-CorV inactivated vaccine has been widely used in 93 countries. In order to understand deeply the protective mechanism of inactivated vaccine, which retains all antigenic components of live virus, the analysis of humoral responses triggered by multiple proteins is necessary. In this research, antibody responses were generated with 6 selected recombinant proteins and 68 overlapping peptides that completely covered SARS-CoV-2 nucleocapsid (N) protein in 254 healthy volunteers vaccinated with BBIBP-CorV. As a result, antibody responses to the receptor binding domain (RBD), N, and non-structural protein 8 (NSP8) were induced following immunization by BBIBP-CorV. The antibody responses detected in donors after the 2^nd^ dose vaccination can be maintained for about 6 months. Moreover, specific antibody levels can be restored after the boosting vaccination measured by ELISA. Furthermore, the level of SARS-CoV-2 specific IgG response is independent of age and gender. Moreover, N_391-408_ was identified as a dominant peptide after vaccination of BBIBP-CorV through peptide screening. Understanding the overview of humoral reactivity of the vaccine will contribute to further research on the protective mechanism of the SARS-CoV-2 inactivated vaccine and provide potential biomarkers for the related application of inactivated vaccine.

## Introduction

Coronavirus disease 2019 (COVID-19), caused by severe acute respiratory syndrome coronavirus 2 (SARS-CoV-2), has become a serious threat to public health (Zhou et al., 2020). So far 621 million cases of COVID-19 have been diagnosed, with 6.56 million deaths (https://coronavirus.jhu.edu/map.html; Dong et al., 2020). The SARS-CoV-2 genome encodes 16 non-structural proteins (NSP1-16), nine accessory proteins (ORF3a, 3b, 6, 7a, 7b, 8, 9a, 9b, and 10), and four structural proteins including spike (S), nucleocapsid (N), envelope (E), and membrane (M; Wu et al., 2020). The S protein mediates virus entry into cells, and the receptor binding domain (RBD) on the S1 subunit plays an important role in virus recognition and binding to cells (Ge et al., 2013; Hoffmann et al., 2020; Jiang et al., 2020; Lan et al., 2020; Wrapp et al., 2020). Meanwhile, most of the neutralizing epitopes found are located in RBD (Piccoli et al., 2020; Robbiani et al., 2020).

On a global scale, herd immunity through vaccination is the best way to return to normal life (Jeyanathan et al., 2020; Dai and Gao, 2021). According to statistics,^1^ 47 kinds of vaccines are approved for use. These vaccines can be divided into several groups, namely, subunit vaccines (Toback et al., 2021; Xia et al., 2021), RNA/DNA vaccines (Baden et al., 2021; Thomas et al., 2021), viral vector vaccines (Falsey et al., 2021; Halperin et al., 2022), and inactivated viral vaccines (Wang et al., 2020; Ella et al., 2021; Medeiros-Ribeiro et al., 2021). The inactivated virus vaccine is considered as one of the best options due to its low cost, high safety, high efficiency, and high feasibility (Ni et al., 2020). There are currently 11 kinds of inactivated SARS-CoV-2 vaccines approved for use, including Sinopharm BBIBP-CorV (Wang et al., 2020; Xia et al., 2021), SinoVac CoronaVac (Tanriover et al., 2021), and Bharat Biotech BBV152/Covaxin (Ella et al., 2021) et al.

The BBIBP-CorV is the most widely used inactivated SARS-CoV-2 vaccine in the world, which has been approved in 93 countries. The SARS-CoV-2 strain of BBIBP-CorV (19NCOV-CDC-Tan-HB02, HB02) was isolated from the bronchoalveolar lavage sample of a hospitalized patient at the beginning of the outbreak. The BBIBP-CorV was inactivated by β-propionolactone treatment and prepared with aluminum hydroxide as adjuvant (Wang et al., 2020). A randomized double-blind phase III clinical trial conducted in the United States and Bahrain demonstrated that the BBIBP-CorV showed a good protective efficacy. The efficacy for BBIBP-CorV was 78.1%(Al Kaabi et al., 2021).

The immune responses of SARS-CoV-2 have been well studied in infected and vaccinated individuals. At present, the humoral immunity research of inactivated vaccines mainly focus on five aspects: immunogenicity and neutralizing activity (Chan et al., 2021; Yadav et al., 2021), neutralizing activity against mutant strains (Cao et al., 2021; Acevedo et al., 2022; Bartsch et al., 2022; Yu et al., 2022), immunogenicity in patients with underlying diseases (Medeiros-Ribeiro et al., 2021; Aikawa et al., 2022; Netto et al., 2022; Szebeni et al., 2022), dynamic changes of neutralizing antibodies (Sauré et al., 2022), and neutralizing activity after booster vaccination (Ai et al., 2022a; Cheng et al., 2022; Costa Clemens et al., 2022). However, the humoral immune response has not been fully understood. Inactivated vaccines retain all the antigenic components of live viruses, as such, it is also important to understand the specific antibody responses to multiple SARS-CoV-2 proteins. Nevertheless, to our knowledge, the study focused on the non-S protein antibody response is limited.

This study was conducted to understand the IgG and IgM responses triggered by inactivated SARS-CoV-2 vaccine, BBIBP-CorV. Serum samples from 254 healthy individuals vaccinated with the homologous BBIBP-CorV inactivated vaccine were analyzed. Two structural proteins (RBD, and N proteins) and four non-structural proteins (NSP1, NSP5, NSP7, and NSP8) were initially selected for antibody response analysis. We chose RBD because its antibody titer is correlated with antibody neutralization ability (Ma et al., 2021). The N protein was selected because of its strong immunogenicity and high homology between different beta coronavirus and SARS-CoV-2 variants (Jiang et al., 2020). As for the four selected non-structural proteins, they are representatives of ORF1a/b polyproteins involved in viral replication, translation, and protein splicing (Dai et al., 2020; Yin et al., 2020; Lapointe et al., 2021), and their immunogenicity were also reported (Jiang et al., 2020; Le Bert et al., 2020; Ong et al., 2020; Zolfaghari Emameh et al., 2020). The dominant antibody responses after vaccination at protein and peptide levels were identified. Furthermore, the dynamics of SARS-CoV-2-specific antibody response after vaccination and the relationship between antibody response with age and gender were analyzed. Moreover, an immunodominant linear B cell epitope on the N protein was identified and this epitope may have the potential to be a complement to serological detection and vaccine efficacy evaluation.

## Materials and methods

### Ethics statement

This study was approved by the Ethics Review Committee of the Eighth Affiliated Hospital of Sun Yat-sen University (reference 2021-005-01). Under the principles of the Helsinki declaration, the written informed consent of the participants was obtained.

### Human samples

Serums were collected from 254 volunteers who had been examined at the Eighth Affiliated Hospital of Sun Yat-sen University since July 2022. After coagulation, serum was collected in an IMPROVE Vacutainer SST tube. The serum was centrifuged at 3,500 rpm for 10 min and was separated before preservation at −80°C. The demographic and clinical characteristics of the volunteers were shown in Table 1.

**Table 1:** Demographic and clinical data of participants.

	Unvaccinated grounp (*n* = 31)	1^st^ dose group (*n* = 31)	2^nd^ dose group					3^rd^ dose group (*n* = 31)
			10-30d (*n* = 28)	31-60d (*n* = 31)	61-90d (*n* = 34)	91-180d (*n* = 40)	>180d (*n* = 28)	
Demographics								
Age, years	27(24,36)	27(23,37)	27(24,39)	26(23,31)	28(24,35)	32(25,40)	31(26,40)	32(28,34)
Sex								
Male	15	14	15	16	17	20	14	15
Female	16	17	13	15	17	20	14	16
Days after vaccination	/	28(23,34)	28(22,29)	41(34,56)	72(66,79)	117(107,132)	217(212,224)	72(22,79)

Data represented as Median (IQR).

### Preparation of recombinant protein

The nucleic acid sequences encoding the selected proteins in the SARS-CoV-2 reference sequence NC_045512.2 were downloaded from NCBI, and delivered to the MiaoLing Plasmid Platform (Wuhan, China) to construct expression plasmids using PET-28a as a vector and transferred into *E. coli* BL-21. After verification by sequencing, induction expression of recombinant protein was carried out. *E. coli* (1:100) was added to LB medium (Kana-resistance) and was shaken until OD600nm was 0.8. IPTG (Sigma-Aldrich, #I6758) was added and continued culture for 3 h. It’s confirmed by electrophoresis that proteins expressed in the supernatant after ultrasound. Then, the ultrasonic supernatant was purified by AKTA pure (GE) system. The ultrasonic supernatant was transferred to Ni-NTA prepacked chromatographic column (Sagon Biotech, #C600792). The eluent was a Tris–HCl buffer solution containing 250 mM imidazole. Then an ultrafiltration tube (Millipore, #UFC9010 and #UFC9003) was used for eluent concentration and buffer replacement. Protein purity was determined by SDS-PAGE electrophoresis. The concentration of proteins was determined by BCA (Beyotime, #P0012S). N, NSP1, NSP5, NSP7, and NSP8 proteins used in this study were prepared in our laboratory (see Supplementary Figure 1) and RBD was purchased from SinoBiological (#40592-V08b). Proteins were confirmed by mass spectrometry (BiotechPack, Beijing, China).

### Peptide synthesis and peptide library

The NCBI entry number used to design the SARS-CoV-2 nucleocapsid linear peptide sequence is YP_009724397.2. The principle of overlapping peptides design: offset six amino acids and overlap 12 amino acids. The N protein peptide library (ChinaPeptides, Shanghai, China) consists of sixty-eight 18-mer overlapping peptides (see Supplementary Table 1), which can be used individually or as a set. Eight to nine peptides are combined to form a peptide pool (see Supplementary Table 2). The lyophilized individual peptide is dissolved at 20 μM in DMSO (Sigma-Aldrich, #D8418). The original solution was obtained and stored at −80°C.

### Detection of SARS-CoV-2 specific antibodies

An indirect ELISA assay for the detection of IgG and IgM responses of selected proteins and peptides was performed according to the following instructions. In brief, ELISA plates (ThermoFisher, #446469) were coated with the recombinant proteins (a 2 μg/l carbonate solution) or peptides (a 5 μM/l solution of individual peptide or pool) at 4°C overnight. ELISA plates were blocked with 1% BSA-PBST (0.05% Tween-20) for 2 h at 37°C after washing 3 times with PBST (0.05% Tween-20). 100 μl serum samples (for protein-ELISA, 1:100 dilution; for peptide-ELISA, 1:25 dilution) were added to each well and incubate for 1 h at 37°C before washing 5 times. Next, 100 μl HRP labeled goat Anti-human IgG (ZSGB-Bio, #ZB-2304) or HRP labeled goat anti-human IgM (Jackson ImmunoResearch, #109–035-043; for protein-ELISA, 1:10000 dilution; for peptide-ELISA, 1:5000 dilution) were used for detection of antibodies. In total, 100 μl TMB (TIANGEN, #PA107) was used for a 10 min development and was stopped by addition of 100 μl of stop solution (Sangon Biotech, #E661006), prior to absorbance measurements. OD450nm value was measured with the microplate reader (ThermoFisher).

### Sequence alignment and data visualization

N protein sequences of seven human coronavirus-infected species (accession number: YP_009724397.2 AAP33707.1 YP_00904721.1 YP_009555244.5 1.1 YP_173242.1) YP_003771.1 and NP_073556.1) were downloaded from NCBI database. N protein sequences of Six VOC and four omicron variants were downloaded from the COVID-19 database.^2^ The Mafft algorithm with default values was used for comparison in Jalview 2.11.1.4. Structural data of SARS-CoV-2 N protein were obtained from I-TASSER and visualized with PyMOL (Schrodinger, version 2.3.2).

### Quantification and statistical analysis

A positive control has been selected out to account for plate-to-plate variations. Corrected OD450nm values of samples were normalized to the positive control, and background signals were subtracted. Signal Intensity was defined as the test value minus blank value in OD450nm. Two independent repeated experiments were performed for each sample. The dominant peptide was the one that tested the highest Corrected OD450nm value. Dominant Frequency (%) was calculated as dominant peptide (pool) frequency divided by total frequency. Student’s *t*-test, Mann–Whitney test, ordinary one-way analysis of variance (ANOVA), Kolmogorov–Smirnov test, and Pearson correlation were analyzed by GraphPad Prism 9.0. The hypothesis test was two-sided, and we considered the *p*-value <0.05 to be significant.

## Results

### Two complete doses of BBIBP-CorV induced robust antibody responses to nucleocapsid, besides RBD protein

254 volunteers were recruited and serum samples were collected. Among them, 31 participants were unvaccinated. The left 31, 161 and 31 donors were vaccinated by BBIBP-CorV post the 1^st^, 2^nd^, and 3^rd^ dose, respectively. For the 2^nd^ dose group, volunteers were divided into five subgroups according to the time post vaccination. The demographic and clinical characteristics of the volunteers were summarized in Table 1. Five proteins were prepared in our laboratory with purity≥85% (Supplementary Figure 1) and RBD was purchased from a company with purity≥95%.

**Figure 1:**
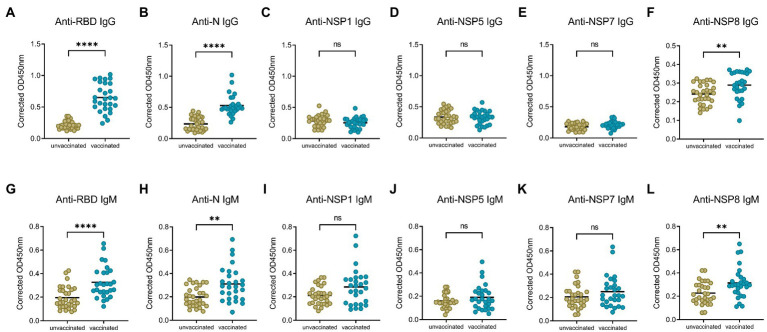
SARS-CoV-2 specific antibody responses elicited by two doses of BBIBP-CorV vaccination. **(A–F)** IgG responses against SARS-CoV-2 proteins, including the RBD **(A)**, N **(B)**, NSP1 **(C)**, NSP5 **(D)**, NSP7 **(E)** and NSP8 **(F)**. **(G–L)** IgM responses to the RBD **(G)**, N **(H)**, NSP1 **(I)**, NSP5 **(J)**, NSP7 **(K)**, NSP8 **(L)**. Serums were from the unvaccinated group (*n* = 31) and the 10–30 days post the 2^nd^ dose group (*n* = 28). Corrected OD450nm values of samples were normalized to a positive control, and background signals were subtracted. Value of *p* was calculated by the two-sided Student’s *t*-test or Mann–Whitney test. *p < 0.05, **p < 0.01, ***p < 0.005, ****p < 0.001, ns represents not significant.

To obtain a profile of SARS-CoV-2 specific antibody response in vaccinated individuals, we performed indirect ELISA assays using six SARS-CoV-2 recombinant proteins (two structural proteins and four non-structural proteins). The IgG and IgM antibody responses to different proteins were detected and compared between the unvaccinated group and the 10–30 days post the 2^nd^ dose group. In terms of IgG response (Figures 1A–F), the IgG responses of RBD, N, and NSP8 in the vaccinated group were significantly higher than those in the unvaccinated group (3.03-,2.23- and 1.20-fold, respectively), while there were no significant difference in the responses of NSP1, NSP5, and NSP7 between two groups. As for IgM responses, the results were similar to IgG (Figures 1G–L), the IgM responses of RBD, N, and NSP8 in the vaccinated group were significantly higher than those in the unvaccinated group (1.67-, 1.56- and 1.39-fold, respectively), whereas the IgM responses of NSP1, NSP5, and NSP7 showed any significant change after BBIBP-CorV vaccination. Those results are consistent with the trends of naturally infected individuals.(Jiang et al., 2020) Notably, some individuals had significantly elevated levels of NSP1, NSP5, or NSP7 IgM response (Figures 1I–K). In general, RBD, N, and NSP8 all induced significant antibody responses, among which the stimulating effects of RBD and N protein were more substantial.

### The 1st dose vaccination of BBIBP-CorV induced slight antibody responses to RBD protein, in contrast, robust responses to nucleocapsid

**Figure 2:**
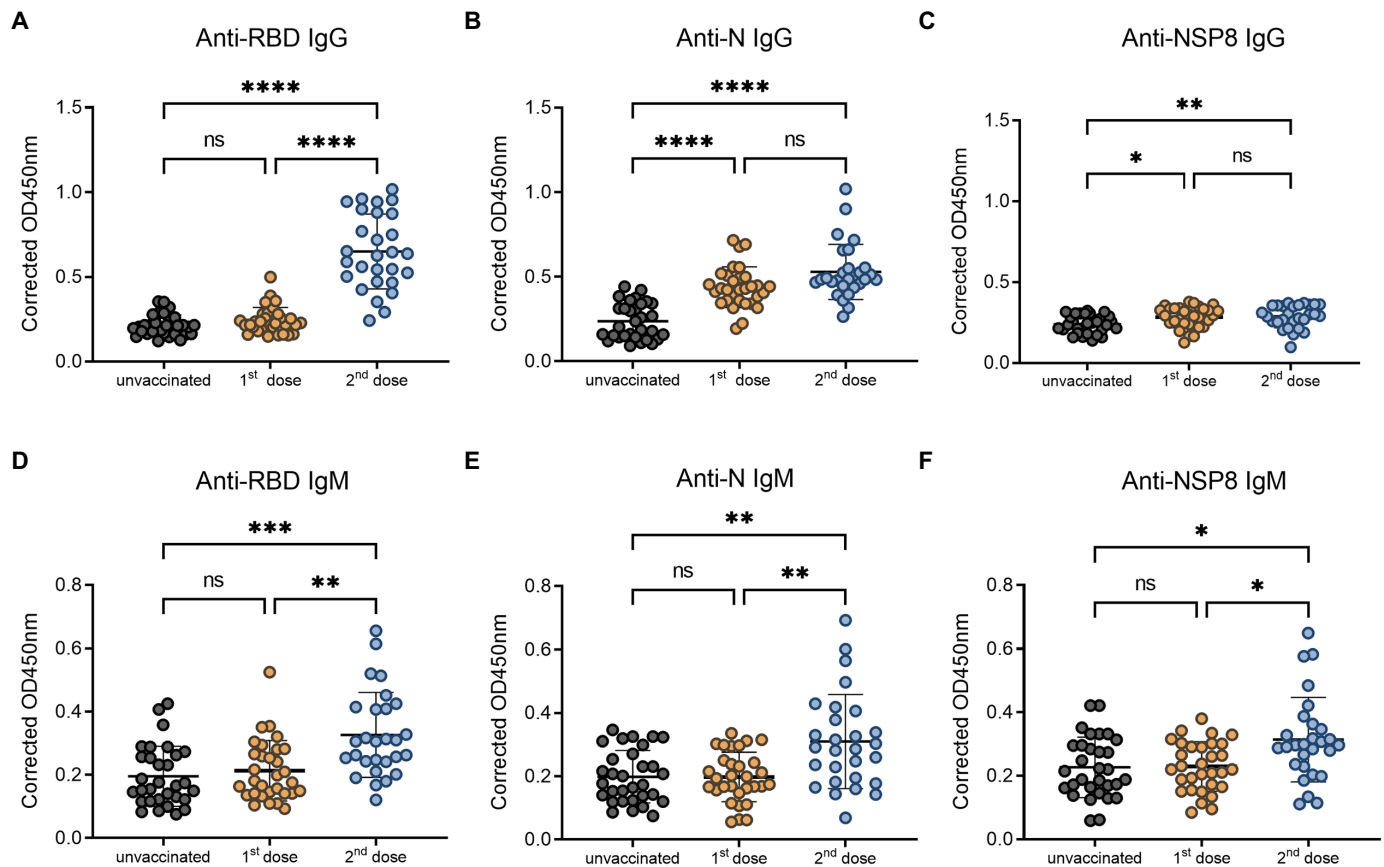
SARS-CoV-2 specific antibody responses after different doses of BBIBP-CorV. **(A–C)** IgG responses to RBD protein **(A)**, N protein **(B)**, and NSP8 protein **(C)**. **(D–F)** IgM responses to RBD protein **(D)**, N protein **(E)**, NSP8 protein **(F)**. Serum samples were from the 1^st^ dose vaccination group (*n* = 31), and 10–30 days post the 2^nd^ dose vaccination group (*n* = 28) with unvaccinated group (*n* = 31) as control. Value of *p* was calculated by one-way analysis of variance (ANOVA) or Kolmogorov–Smirnov test.

To compare the change of antibody levels after the 1^st^ and 2^nd^ dose of vaccination, serum samples from the 1^st^ dose immunization group, and 10–30 days post the 2^nd^ dose of vaccination group were analyzed, with the IgG and IgM responses of unvaccinated group investigated as control. Specific IgG and IgM antibody levels for RBD, N, NSP8 among these three groups were detected *via* indirect ELISA assay. For IgG antibody (Figures 2A–C), the 1^st^ dose of vaccination induced a significant N and NSP8 IgG response (1.85-, 1.18-fold, respectively), but no RBD IgG response was found (1.13-fold). After the 2^nd^ dose of vaccination, although there was no statistically significant increase in N and NSP8 IgG levels compared with the 1^st^ dose (2.24-, 1.20-fold, respectively), but still showed increasing trends. Besides, some individual’s IgG against N protein have increased considerably. Meanwhile RBD-specific IgG antibody increased significantly (3.03-fold) after the 2^nd^ dose vaccination. For specific IgM responses (Figures 2D–F), the 1^st^ dose vaccination did not elicit statistically significant higher antibody levels (1.09-, 1.00-, 1.01-fold, respectively), which was further elevated by the second one (1.67-, 1.56-, 1.39-fold, respectively). Overall, elevated N and NSP8 IgG antibody response were observed after the 1^st^ dose, boosted antibody levels of anti-N, -NSP8 IgM, and anti-RBD IgG/IgM were observed post the 2^nd^ dose vaccination. These results suggest that secondary vaccination strategy of BBIBP-CorV is necessary.

### Nucleocapsid-specific antibody responses persisted for more than 6 months

**Figure 3:**
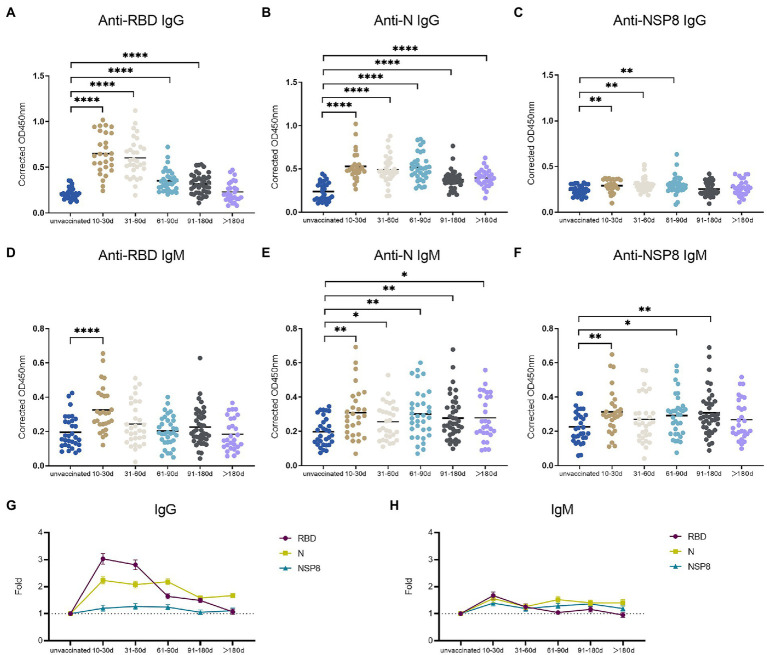
SARS-CoV-2 specific antibody responses after the 2^nd^ dose of vaccination. **(A–C)** SARS-CoV-2 specific IgG responses to RBD protein **(A)**, N protein **(B)**, and NSP8 **(C)**. **(D–F)** SARS-CoV-2 specific IgM responses to RBD protein **(D)**, N protein **(E)**, and NSP8 **(F)**. Serums were collected from 10–30 days (*n* = 28), 31–60 days (*n* = 31), 61–90 days (*n* = 34), 91–180 days (*n* = 40) and >180 days (*n* = 28) group post the 2^nd^ dose vaccination with unvaccinated group (n = 31) as control. The *p*-value was calculated by Student’s *t*-test or Mann–Whitney test. Only differences from the unvaccinated group were shown. **(G,H)** Summary dynamics of specific IgG **(G)** and IgM **(H)** responses induced by different SARS-CoV-2 antigens after the 2^nd^ dose vaccination with unvaccinated group as control. The X-axis represents the period after two doses of vaccine were administered. The Y-axis shows the ratio to the mean of the unvaccinated group shown as mean ± SEM.

To determine the dynamics of SARS-CoV-2 specific antibody response, we analyzed serum samples from the unvaccinated group, 10–30 days, 31–60 days, 61–90 days, 91–180 days and >180 days subgroups of the post 2^nd^ dose of vaccination. In this study, the RBD-, N-, and NSP8-specific IgG and IgM responses were detected by indirect ELISA method. The specific IgG and IgM responses against those 3 antigens showed the same trend as antibody levels increased to a plateau and then decreased over time after the 2^nd^ dose vaccination of BBIBP-CorV. In specific IgG antibody response (Figures 3A–C), RBD antibody response reached the peak at 10-30d (3.03-fold) post two-dose vaccination. The response level remained high within 180 days after two-dose vaccination especially within 60 days. The level of anti-RBD IgG began to decrease after 60 days and decreased assemble to pre-vaccination after 180 days. N protein antibody response peaked at 10-30d (2.24-fold) post vaccination, and began to decline after 90 days, but still slightly higher than the pre-vaccination level after 180 days. NSP8-specific IgG levels peaked at 31-60d (1.27-fold) post two-dose vaccination and then began to decline after 90 days. Among specific IgM antibody responses, anti-RBD IgM only appeared at the initial 10-30d after vaccination (1.67-fold), and there was no statistical difference in response level after 30 days compared with pre-vaccination (Figure 3D). Meanwhile anti-N, and -Nsp8 IgM responses persisted for longer (Figures 3E,F). Generally, RBD induced the highest level of specific IgG and IgM response, followed by N protein, and NSP8 was the lowest (Figures 3G,H). Nevertheless, the response duration of N protein was the longest, followed by RBD and NSP8 (Figures 3A–C). In summary, two-dose of BBIBP-CorV immunization produced a strong IgG antibody response with the highest RBD response level and the longest N protein duration.

### Boosting vaccination of BBIBP-CorV are necessary to restore antibody response levels

**Figure 4:**
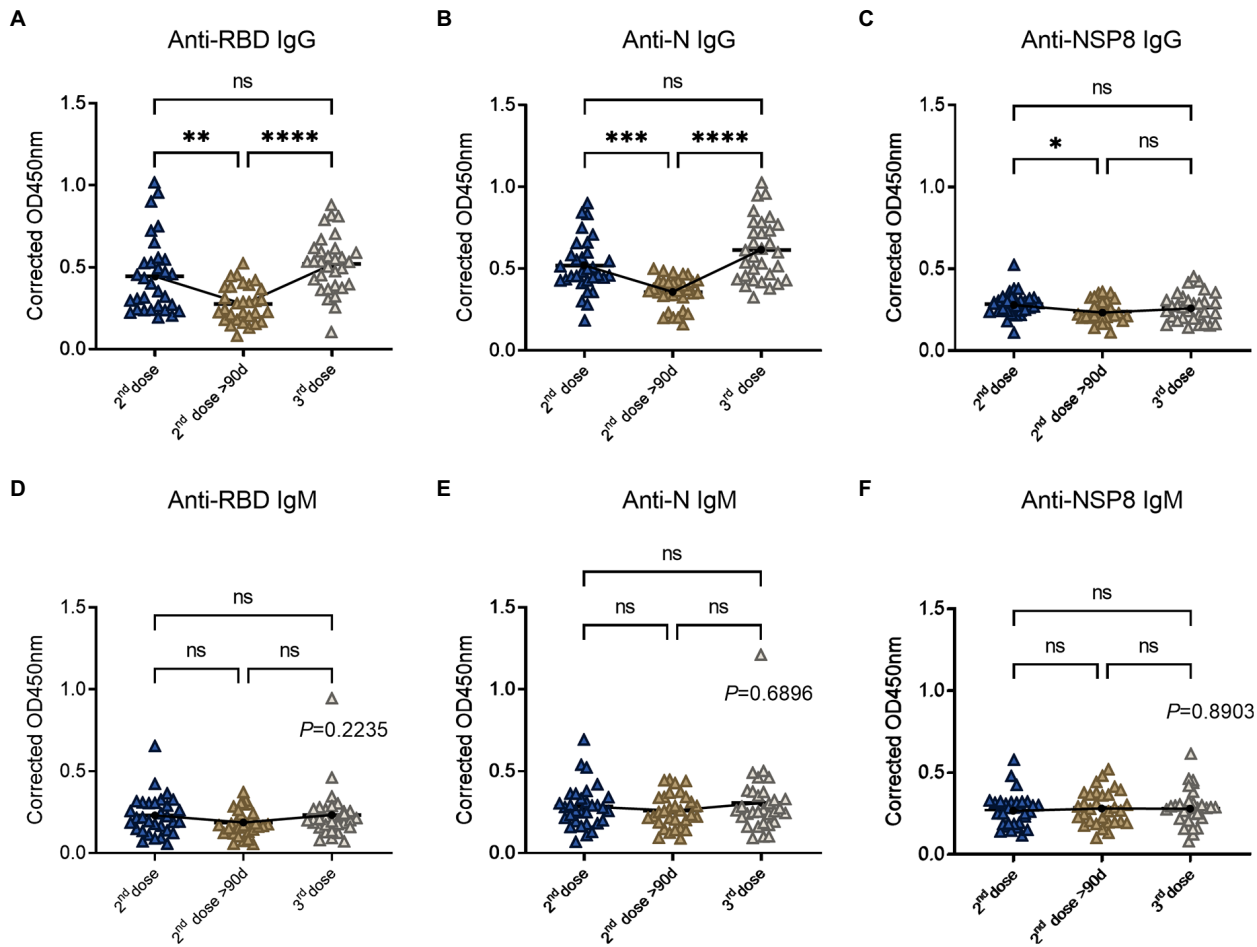
SARS-CoV-2 specific antibody responses after the 3^rd^ dose of vaccination. **(A–C)** Specific IgG responses to RBD protein **(A)**, N protein **(B)** and NSP8 **(C)** post the 3^rd^ dose vaccination with two 2^nd^ dose vaccination subgroups as control. **(D–F)** Specific IgM responses to RBD protein **(D)**, N protein **(E)**, NSP8 **(F)** post the 3^rd^ dose vaccination with two 2^nd^ dose vaccination subgroups as control. Serum samples were from the 10–90 days subgroup (*n* = 31), >90 days subgroup (*n* = 31) post the 2^nd^ dose vaccination and the 3^rd^ dose vaccination group (*n* = 31). The *p*-value was calculated by one-way analysis of variance (ANOVA) or Kolmogorov–Smirnov test.

To determine the changes of SARS-CoV-2 specific antibodies after the 3^rd^ dose vaccination, we analyzed 93 volunteers’ samples from the 10-90d group, > 90d group after the 2^nd^ dose vaccination, and the 3^rd^ dose vaccination group. Serum samples were tested for specific IgG and IgM responses of the 6 selected proteins. In terms of specific IgG antibody response (Figures 4A–C), RBD-specific antibody (2.42-fold) increased after the 3^rd^ dose compared with the 2^nd^ dose >90d group (1.29-fold), which was equivalent to the degree of the 2^nd^ dose group (2.07-fold) and showed an increasing trend. The laws of evolution for N-specific IgG response were the same as that of RBD. Three-dose of the BBIBP-CorV also boosted N-specific IgG response (2.60-fold) compared to the 2^nd^ dose >90d group (1.51-fold). NSP8-specific IgG increased after the 3^rd^ dose, but the difference was not statistically significant. For specific IgM response (Figures 4D–F), there was no statistical difference among three groups, which may be caused by the immunological characteristics of IgM. In conclusion, RBD-and N-specific IgG post the 3^rd^ dose vaccination which equivalent to the degree of the 2^nd^ dose group showed statistical differences to the 2^nd^ dose >90d group. Above results indicate the effectiveness and necessity of the boosting vaccination strategy.

### The IgG response to nucleocapsid is unrelated to age and gender in vaccinated volunteers

**Figure 5:**
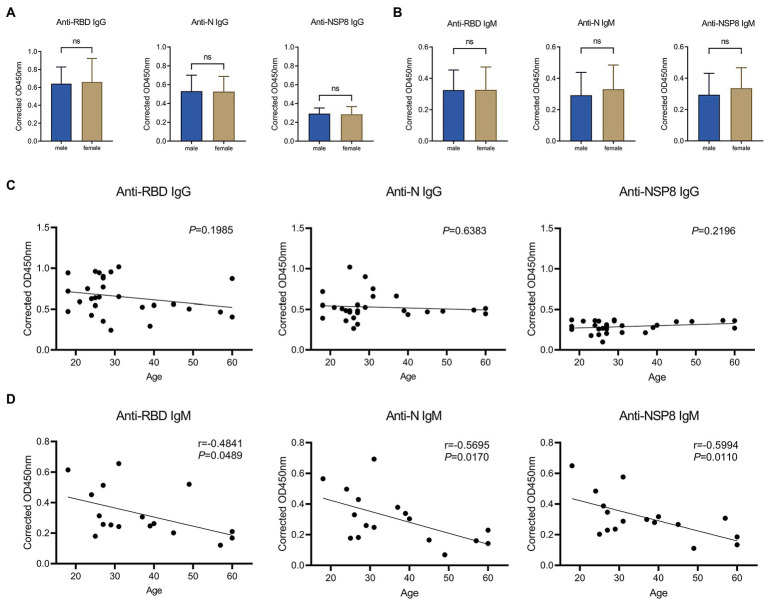
Correlation of SARS-CoV-2 specific antibody responses to gender or age. **(A)** SARS-CoV-2 specific IgG response of male vs. female to the RBD, N, and NSP8. Serums were collected from the 10–30 days after the 2^nd^ dose vaccination group (*n* = 28). **(B)** IgM responses of male vs. female to the RBD, N protein, and NSP8. Serums were collected from the 10–30 days after the 2^nd^ dose vaccination group (*n* = 28). The *p*-value was calculated by the Student’s *t*-test or Mann–Whitney test. **(C)** Correlation of RBD-, N-, and NSP8-specific IgG responses to age. Serums were collected from the 10–30 days after the 2^nd^ dose vaccination group (*n* = 28). **(D)** Correlation of RBD-, N-, and NSP8-specific IgM responses to age. Serums were collected from the 10–30 days after the 2^nd^ dose vaccination group (*n* = 28). A correlation test was conducted using Pearson correlation.

A comparative analysis was conducted to assess the difference in antibody response between males and females after complete vaccination. In terms of IgG responses (Figure 5A), there were no significant differences in RBD, N, and NSP8 responses between males and females in 28 volunteers from the 10-30d after the 2^nd^ dose vaccination group. In terms of IgM response (Figure 5B), no significant difference was found between males and females in 28 volunteers from the 10-30d after the 2^nd^ dose vaccination group. It was concluded that the specific antibody responses post vaccination of BBIBP-CorV was not correlated with gender. To assess whether antibody responses related to age, we performed a Pearson correlation analysis between SARS-CoV-2 specific antibody responses with age. In terms of IgG response (Figure 5C), among 28 volunteers from the 10-30d group after the 2^nd^ dose, results show no correlation of the RBD-, N-, and NSP8-specific IgG responses with age. As for the IgM response (Figure 5D), correlation analysis was performed on 28 volunteers from the 10-30d group after the 2^nd^ dose, RBD-, N-, and NSP8-specific IgM responses were negatively correlated with age, with correlation coefficients being −0.48, −0.57 and − 0.60, respectively. In general, the SARS-CoV-2 specific IgG and IgM responses post vaccination of BBIBP-CorV were not correlated with gender. SARS-CoV-2 specific IgG response was not correlated with age, while IgM response was surprised negatively correlated with age.

### The dominant epitope to nucleocapsid located in N_391-408_ after vaccination

**Figure 6:**
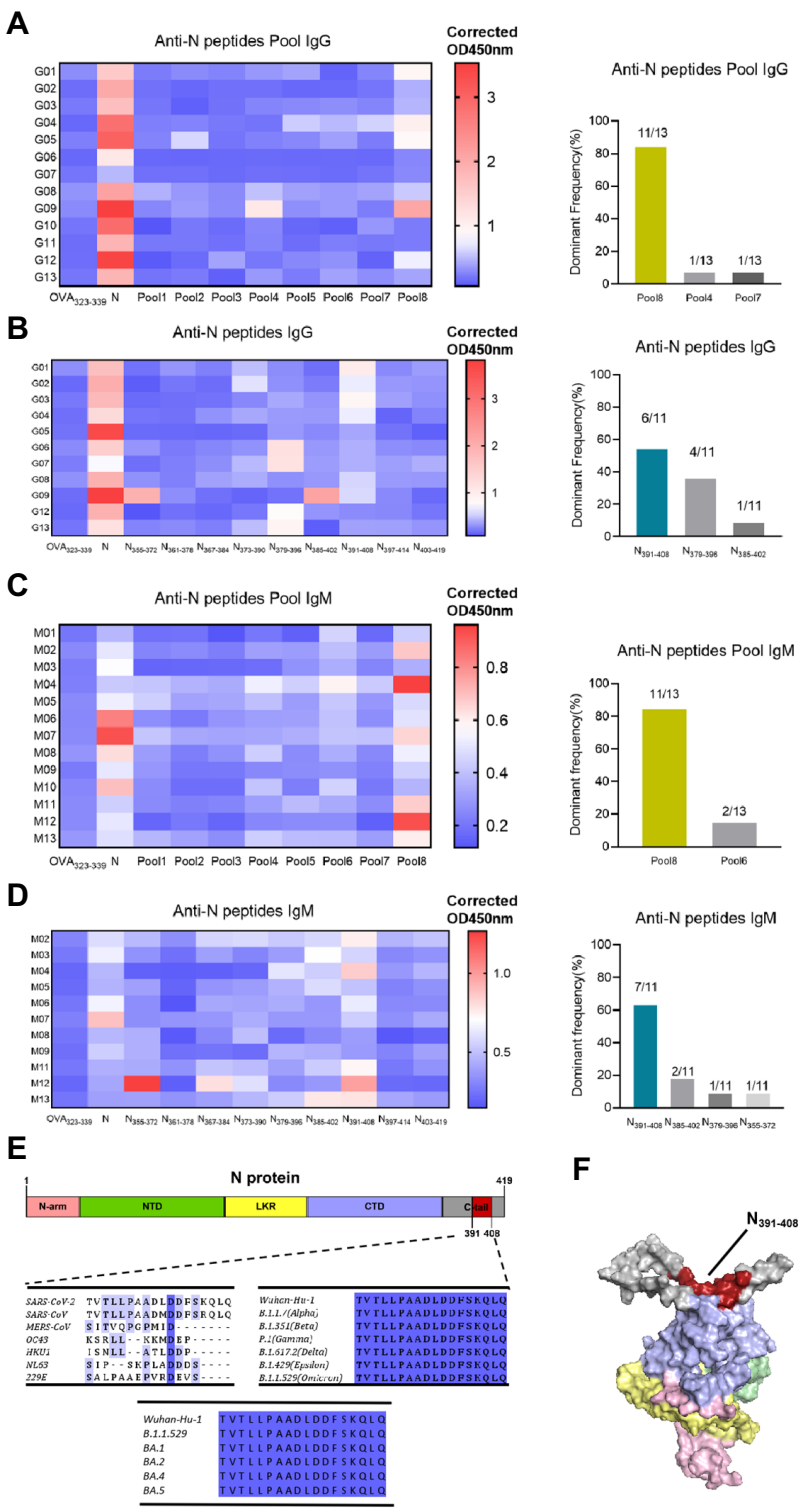
Identification of dominant specific linear B-cell epitope on N protein with vaccinated volunteer serums. **(A)** Serum samples (1:25 dilution) were tested in a peptide-based ELISA of peptide pools covering SARS-CoV-2 N protein for IgG. Each pool consists of 8–9 18-mer overlapping peptides. Antibody response levels were detected by indirect ELISA assay with OVA_232-339_, N protein as the negative and positive control, respectively. OD450nm values are presented in a heatmap, with blue and red colors denoting low and high OD450nm values, respectively. The dominant frequency of different pools also had been counted in the histogram. Thirteen individuals with the highest N-specific IgG response levels were selected. Dominant Frequency (%) was calculated as dominant peptide (pool) frequency divided by total frequency. **(B)** Individual peptide of the dominant peptide pools in **(A)** were tested to determine the IgG dominant peptide. Serums were from pool8-dominant volunteers (*n* = 11). The dominant frequency of different peptides also had been counted in the histogram. **(C)** IgM responses to N protein peptide pools. Serums were from 13 high protein-ELISA OD value vaccinated volunteers. **(D)** IgM responses to the individual peptide of pool8. Serum were from pool8-dominant volunteers (*n* = 11). **(E)** Dominant epitope sequence alignment among 7 human-infected coronaviruses and SARS-CoV-2 variant strains. The depth of amino acid blue background indicates the degree of homology. **(F)** Localization of N_391-408_ on the 3-D structure of N protein. Red represents the dominant epitope N_391-408_. N-arm, NTD, LKR, CTD, and C-tail are shown as pink, green, yellow, purple, and gray, respectively.

Considering that N-specific antibody is characterized by high titer and durability, we identified the dominant epitope of N protein in this research. To obtain a global map of SARS-CoV-2 N-specific antibody response in vaccinated population, sixty-eight 18-mer peptides were synthesized by the principle of stepping six amino acids and overlapping 12 amino acids, covering the entire length of N protein (419aa). Thirteen individuals whose N-specific IgG or IgM responses level are above cut-off value (x̄ + 3σ) were tested, respectively. These peptides were divided into eight peptide pools, each containing 8–9 peptides. The indirect ELISA assay was performed to detect peptide-specific IgG response (Figure 6A). As a result, at the peptide pools level, several peptide pools showed serum reactivity, such as Pool8, Pool4, Pool7, Pool5, Pool6, and Pool2. And Pool8 showed a significant dominance in these pools. Then we further screened out the dominant epitope of Pool8 based on the population with Pool8-dominant response volunteers (Figure 6B). At the individual peptide level, several peptides showed serum reactivity, such as N_373-390_, N_379-396_, N_385-402_, N_391-408_. And N_391-408_ showed a significant dominance in these peptides. Similar to IgG response, IgM response also displayed a dominance in Pool8 and N_391-408_ (Figures 6C,D). In summary, SARS-CoV-2 N-specific IgG and IgM response after vaccination of BBIBP-CorV were concentrated on N_391-408_ peptide (TVTLLPAADLDDFSKQLQ). We compared the sequences of N_391-408_ to that of all coronaviruses known to infect humans, the immune dominant N_391-408_ were highly specific between species (except SARS-CoV). By comparing the conservation degree of N_391-408_ among the Variants of Concern (VOC), N_391-408_ was 100% conserved among different mutant strains. It’s worthy noting that N_391-408_ was also conserved among currently epidemic omicron variants (Figure 6E). The spatial localization of N_391-408_ on three-dimensions structure of N protein (Figure 6F) showed that the dominant sequence was located on the surface of the N protein, which also provided a reasonable explanation for the dominant response of N_391-408_.

## Discussion

Vaccination is one of the best strategies to contain SARS-CoV-2 epidemic. Among the various SARS-CoV-2 vaccines approved for use, the BBIBP-CorV inactivated vaccine has been widely used in 93 countries. Nevertheless, the immune response of non-S protein after vaccination of inactivated vaccines is slightly less studied. This study was conducted to understand the IgG and IgM responses triggered by an inactivated vaccine, BBIBP-CorV, in a long term.

At the protein level, results imply that SARS-CoV-2 specific IgG and IgM of RBD, N and NSP8 were induced by BBIBP-CorV in healthy populations compared with unvaccinated populations. Using inactivated vaccines have the characteristic of a broader immune response compared to other vaccine platforms that use only Spike protein. Strong antibody responses against S and N proteins also detected in COVID-19 patients (Jiang et al., 2020). As a result, S and N proteins have been widely used as antigens for diagnosis of COVID-19. We confirm that antibody responses against RBD protein were strongest. In contrast, antibody responses against N protein play a dominant role after SARS-CoV-2 infection (Jiang et al., 2020). The distinct natures of the inactivated and live viruses may explain this difference. As we all know, RBD-specific antibody titer is correlated with serum neutralization ability in SARS-CoV-2 infected individuals. Similar to previous results from infected individuals, Ma et al. (2021) found that RBD-specific antibody titer is positive correlated with serum neutralizing capacity after vaccination of BBIBP-CorV. Consequently, the significant increase of RBD-specific antibody can be another support to the efficiency of BBIBP-CorV. Though significant differences of anti-NSP1, -NSP5 and -NSP7 IgM level between pre- and post-vaccination were not observed, a few individuals showed apparently elevated antibody levels. Results suggest that the response varied greatly among individuals. Therefore, it is essential for individuals to confirm protection after vaccination. Meanwhile, inactivated vaccines have also been shown to be effective and have good safety in immunocompromised individuals (Medeiros-Ribeiro et al., 2021; Aikawa et al., 2022; Netto et al., 2022; Szebeni et al., 2022).

We believe that inactivated vaccines were able to induce protective antibodies, besides S-specific antibody. The N protein of CoVs has been shown to be involved in host cell mechanisms, such as immune escape interferon inhibition, RNA interference and apoptosis, and cytokine release (Bai et al., 2021). Moreover, a substantially lower risk of reinfection with SARS-CoV-2 was reported among health care workers with anti-N antibodies than among those who were seronegative. And the level of IgG antibody against N protein was related to viral clearance (Ren et al., 2021). The role of anti-N IgG antibodies in providing protective antiviral immunity is currently unknown. NSP8, which composes the coronavirus polymerase acting as a co-factor (Wang et al., 2020), plays a role in viral RNA synthesis (Yin et al., 2020). A previous study reported that NSP8-specific IgG was induced after SARS-CoV-2 infection (Lei et al., 2022). Moreover, high level of NSP8-specific IgG response increased the risk of death and severity of COVID-19 patients (Lei et al., 2022). These IgG responses might be detrimental during SARS-CoV-2 infections. However, the function of NSP8-specific antibody is still largely unknown. Therefore, the role of NSP8-specific antibody after vaccination by inactivated vaccines need further study. This is of great significance to reveal the protective or damage mechanism of inactivated COVID-19 vaccine.

It is remarkable that the IgG response of RBD is different to that of N protein and NSP8 after different doses. RBD-specific IgG response mainly appears after the 2^nd^ dose, while N-, and NSP8-specific IgG mainly appears after the 1^st^ dose, which indicates that the primary and secondary immunization can trigger different antibody response spectrums and worth further investigation. Because neutralizing antibodies primarily target RBD (Klein et al., 2020; Ni et al., 2020), the significant increase in IgG response after the 2^nd^ dose can be another support of the two-dose vaccination strategy of inactivated vaccine.

There was no difference in RBD, N and NSP8 IgG responses between females and males. Although anti-N and -NSP8 IgM signals were slightly higher in females than in males, the differences were not significant, which is similar to trends observed in convalescent patients (Jiang et al., 2020; Wang et al., 2020). These results suggest that the same vaccination policies can be adopted across genders. A study of BBIBP-CorV also reported no difference between genders (Ma et al., 2021). RBD-, N-, and NSP8-specific IgG responses were not correlated with age after complete two dose vaccination. The above results suggest that the same vaccination policy can be adopted at different ages (18–60 years old). In contrast, Ma et al. (2021) indicate that S-, S1-, and RBD-specific IgG responses were negatively correlated with age after BBIBP-CorV vaccination. This difference may be due to differences in the age distribution of samples. Interestingly, according to the previous studies (Klein et al., 2020; Wang et al., 2020), SARS-CoV-2 specific IgG response is positively correlated with age in cases of SARS-CoV-2 infection. This may be related to different natures of infection and vaccines.

After the 2^nd^ dose vaccination, the IgG response was mainly observed, and the IgM response was low and restored to baseline only take about 4 weeks. IgG levels peaked around 1 month after vaccination (Sauré et al., 2022). Results show that RBD-specific IgG remained high level for about 60 days after two complete doses vaccination, then decreased to baseline after 180 days. Dynamics of RBD-specific antibody can be used as a basis for the necessity and timing of booster vaccination of inactivated vaccine. Previous study found that the average t_1/2_ of RBD-specific IgG was 69 days and remained relatively stable for 6 months after infection, with N-specific IgG dynamics similar to RBD-specific IgG (Dan et al., 2021). Interestingly, N-specific IgG remained higher than the baseline over the observation period of >180d (median, 217 days), suggesting that even though N-specific IgG response was lower than that of RBD at the beginning, however, persisted longer than RBD. Moreover, the level of N-specific IgG exceeds that of RBD-specific IgG after 90 days. Currently approved vaccines are mainly developed based on the SARS-CoV-2 spike or RBD protein. New spike variants, especially RBD multiple mutations, raise concerns about the effectiveness of existing vaccines. Therefore, the development of a vaccine against a highly mutated pandemic SARS-CoV-2 variant with a safe and highly effective broad-spectrum protection against COVID-19 is critical. N protein is highly conserved and may be a potential vaccine target.

After boosting vaccination, SARS-CoV-2 specific IgG response returned to the level of the same period after the 2^nd^ dose, and the response increased slightly, although the difference was not statistically significant. These results suggest that the boosting vaccination is effective, which are similar to previous studies (Ai et al., 2022b; Cheng et al., 2022; Costa Clemens et al., 2022). This may be used as a support for boosting immunization strategies. However, there was no difference in IgM among the three groups, which may be related to the basic immune characteristic of IgM.

Considering that N-specific antibody is characterized by high titer and durability, we identified the dominant epitope of N protein in this research. The overall IgG and IgM responses after two-dose vaccination were mainly concentrated in Pool8, which ranges from amino acids 355 to 419 and belonged to the C-terminal of N protein. The N-terminal domain (NTD) and C-terminal domain (CTD) of SARS-CoV-2 N protein have been analyzed and the CTD owns a dominance compared with NTD (Dinesh et al., 2020; Peng et al., 2020). After screening the dominant peptide pool, we further screened out the dominant epitope of N protein. The most dominant epitope, N_391-408_(TVTLLPAADLDDFSKQL), was located in the C-tail region and set on the surface of N protein, which provided the foundation for its dominant response. To our knowledge, there is no study about the dominant epitope of nucleocapsid after vaccination of SARS-CoV-2 inactivated vaccines. A study based on infected individuals (Qi et al., 2021) mapped the epitopes of N protein by using serum and high-pass scale mapping technology (AbMap), and the region, from amino acids 363 to 416, had a dominance. This region is highly overlapped with the coverage area of the dominant peptide library screened out in our research. Furthermore, the previous researches based on infected individuals identified several epitopes, for example, QQTVTLLPAADLDDFS (Heffron et al., 2021), QKKQQTVTLLPAADL (Schwarz et al., 2021), AADLDDFSKQLQ (Mishra et al., 2021) and TVTLLPAADLDDFSK (Hotop et al., 2022). These epitopes are highly overlapped with the coverage area of the dominant peptide, N_391-408_, screened out in our research.

Antibody assay is a key test for assessing the effectiveness of SARS-CoV-2 vaccination, distinguishing vaccination from natural infection (Ma et al., 2021) and identifying asymptomatic infection. N_391-408_ showed specificity among different viral species (except SARS-CoV) and is 100% conserved among mutant strains. Based on the protective immunity of N protein, we speculate that this epitope may be used as a complement to serological tests and vaccine efficacy evaluation. The localization information, that N_391-408_ is close to N-CTD, suggests that the role and mechanism of the N_391-408_ antibody after vaccination are worthy of further study.

We have to admit that there are several limitations. First, we failed to use paired samples for detection unfortunately. Instead, we tried to reduce the influence of individual differences by increasing the sample size. Second, the application of the dominant epitope needs further experimental verification. Finally, studies on the immunological function and mechanism of dominant epitope antibodies have not been included in this manuscript. Especially to deserve to be mentioned, another team in our group has started related research, and we believe that there will be new progress soon.

In conclusion, by taking advantage of the SARS-CoV-2 protein-based ELISA and N protein peptide-based ELISA, we generated the SARS-CoV-2-specific IgG and IgM responses profile triggered by an inactivated SARS-CoV-2 vaccine, BBIBP-CorV. In this research we detected obvious differences by comparing this profile to those of unvaccinated volunteers. Furthermore, dynamics of SARS-CoV-2-specific antibody response after vaccination were identified and an immunodominant linear B cell epitope on the N protein that may have the potential to be used as a complement to serological detection and vaccine efficacy evaluation.

## Data availability statement

The datasets presented in this study can be found in online repositories. The names of the repository/repositories and accession number(s) can be found in the article/Supplementary material.

## Ethics statement

The studies involving human participants were reviewed and approved by the Ethics Review Committee of the Eighth Affiliated Hospital of Sun Yat-sen University (reference 2021-005-01). The patients/participants provided their written informed consent to participate in this study. Written informed consent was obtained from the individual(s) for the publication of any potentially identifiable images or data included in this article.

## Author contributions

QW and CW drafted the manuscript. All authors edited and reviewed manuscript drafts. LS and AZ were responsible for recruiting volunteers and collecting human samples. FZ and XC provided recombinant proteins. QW and TH provided N protein peptide library. QW, JN, and BL performed immune assays. QW, JN, and YC were responsible for statistical analysis. All authors contributed to the article and approved the submitted version.

## Funding

This project was supported by Shenzhen Science and Technology Program (JCYJ20210324115204012) and Shenzhen Futian District Public Health Research Project (FTWS2020017).

## Conflict of interest

The authors declare that the research was conducted in the absence of any commercial or financial relationships that could be construed as a potential conflict of interest.

## Publisher’s note

All claims expressed in this article are solely those of the authors and do not necessarily represent those of their affiliated organizations, or those of the publisher, the editors and the reviewers. Any product that may be evaluated in this article, or claim that may be made by its manufacturer, is not guaranteed or endorsed by the publisher.

## Footnotes

^1^ https://covid19.trackvaccines.org

^2^ https://ngdc.cncb.ac.cn/ncov/
