# Supplementary material for: The BBIBP-CorV inactivated COVID-19 vaccine induces robust and persistent humoral responses to SARS-CoV-2 nucleocapsid, besides spike protein in healthy adults
Source: Front Microbiol. 2022 Nov 4;13:1008420. doi: 10.3389/fmicb.2022.1008420 (PMC9672472; doi:10.3389/fmicb.2022.1008420)
Supplement: Supplementary file 1 [file Data_Sheet_1.docx]

**Supplementary [materials](C:/Users/wqinj/AppData/Local/youdao/dict/Application/8.10.3.0/resultui/html/index.html#/javascript:;)**


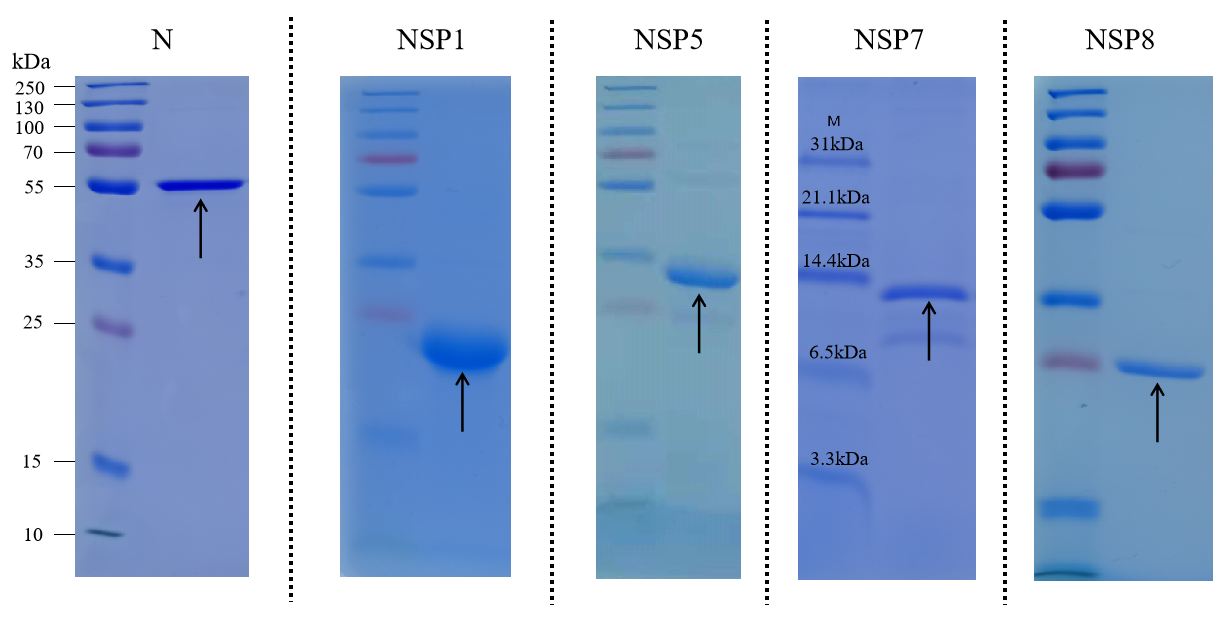


**Supplementary Fig.1 SDS-PAGE band profiles of recombinant proteins prepared by our laboratory.** SARA-CoV-2 N (a), NSP1(b), NSP5(c), NSP7(d), and NSP8(e) protein were made out with purity≥85% determined by SDS-PAGE. The nucleic acid sequence encoding the selected protein from the SARS-CoV-2 reference sequence NC_045512.2 was downloaded from NCBI, and constructed an expression Plasmid using PET-28a as a vector and transferred into *E. coli* BL-21. Recombinant proteins were obtained after induction by IPTG and purification by AKTA pure. Proteins were confirmed by mass spectrometry (BiotechPack, Beijing, China).

**Supplementary Table 1: SARS‐CoV‐2 peptides used in the study**

| SARS-CoV-2：nucleocapsid | | | |
| --- | --- | --- | --- |
| Peptide No | Peptide Sequence | Sequence No | Purity |
| N-1 | MSDNGPQNQRNAPRITFG | N_1-18_ | 93.51% |
| N-2 | QNQRNAPRITFGGPSDST | N_7-24_ | 96.55% |
| N-3 | PRITFGGPSDSTGSNQNG | N_13-30_ | 94.07% |
| N-4 | GPSDSTGSNQNGERSGAR | N_19-36_ | 97.71% |
| N-5 | GSNQNGERSGARSKQRRP | N_25-42_ | 91.50% |
| N-6 | ERSGARSKQRRPQGLPNN | N_31-48_ | 94.74% |
| N-7 | SKQRRPQGLPNNTASWFT | N_37-54_ | 94.18% |
| N-8 | QGLPNNTASWFTALTQHG | N_43-60_ | 93.24% |
| N-9 | TASWFTALTQHGKEDLKF | N_49-66_ | 93.81% |
| N-10 | ALTQHGKEDLKFPRGQGV | N_55-72_ | 93.49% |
| N-11 | KEDLKFPRGQGVPINTNS | N_61-78_ | 97.57% |
| N-12 | PRGQGVPINTNSSPDDQI | N_67-84_ | 92.75% |
| N-13 | PINTNSSPDDQIGYYRRA | N_73-90_ | 93.01% |
| N-14 | SPDDQIGYYRRATRRIRG | N_79-96_ | 94.68% |
| N-15 | GYYRRATRRIRGGDGKMK | N_85-102_ | 93.54% |
| N-16 | TRRIRGGDGKMKDLSPRW | N_91-108_ | 95.11% |
| N-17 | GDGKMKDLSPRWYFYYLG | N_97-114_ | 93.87% |
| N-18 | DLSPRWYFYYLGTGPEAG | N_103-120_ | 95.80% |
| N-19 | YFYYLGTGPEAGLPYGAN | N_109-126_ | 93.71% |
| N-20 | TGPEAGLPYGANKDGIIW | N_115-132_ | 92.24% |
| N-21 | LPYGANKDGIIWVATEGA | N_121-138_ | 91.72% |
| N-22 | KDGIIWVATEGALNTPKD | N_127-144_ | 92.20% |
| N-23 | VATEGALNTPKDHIGTRN | N_133-150_ | 95.84% |
| N-24 | LNTPKDHIGTRNPANNAA | N_139-156_ | 96.52% |
| N-25 | HIGTRNPANNAAIVLQLP | N_145-162_ | 92.35% |
| N-26 | PANNAAIVLQLPQGTTLP | N_151-168_ | 91.50% |
| N-27 | IVLQLPQGTTLPKGFYAE | N_157-174_ | 95.78% |
| N-28 | QGTTLPKGFYAEGSRGGS | N_163-180_ | 92.38% |
| N-29 | KGFYAEGSRGGSQASSRS | N_169-186_ | 94.97% |
| N-30 | GSRGGSQASSRSSSRSRN | N_175-192_ | 92.64% |
| N-31 | QASSRSSSRSRNSSRNST | N_181-198_ | 91.45% |
| N-32 | SSRSRNSSRNSTPGSSRG | N_187-204_ | 92.27% |
| N-33 | SSRNSTPGSSRGTSPARM | N_193-210_ | 91.50% |
| N-34 | PGSSRGTSPARMAGNGGD | N_199-216_ | 97.53% |
| N-35 | TSPARMAGNGGDAALALL | N_205-222_ | 91.98% |
| N-36 | AGNGGDAALALLLLDRLN | N_211-228_ | 96.54% |
| N-37 | AALALLLLDRLNQLESKM | N_217-234_ | 93.56% |
| N-38 | LLDRLNQLESKMSGKGQQ | N_223-240_ | 93.85% |
| N-39 | QLESKMSGKGQQQQGQTV | N_229-246_ | 91.76% |
| N-40 | SGKGQQQQGQTVTKKSAA | N_235-252_ | 92.22% |
| N-41 | QQGQTVTKKSAAEASKKP | N_241-258_ | 95.81% |
| N-42 | TKKSAAEASKKPRQKRTA | N_247-264_ | 93.09% |
| N-43 | EASKKPRQKRTATKAYNV | N_253-270_ | 92.68% |
| N-44 | RQKRTATKAYNVTQAFGR | N_259-276_ | 90.68% |
| N-45 | TKAYNVTQAFGRRGPEQT | N_265-282_ | 91.82% |
| N-46 | TQAFGRRGPEQTQGNFGD | N_271-288_ | 95.15% |
| N-47 | RGPEQTQGNFGDQELIRQ | N_277-294_ | 91.81% |
| N-48 | QGNFGDQELIRQGTDYKH | N_283-300_ | 90.71% |
| N-49 | QELIRQGTDYKHWPQIAQ | N_289-306_ | 93.33% |
| N-50 | GTDYKHWPQIAQFAPSAS | N_295-312_ | 93.98% |
| N-51 | WPQIAQFAPSASAFFGMS | N_301-318_ | 94.73% |
| N-52 | FAPSASAFFGMSRIGMEV | N_307-324_ | 92.19% |
| N-53 | AFFGMSRIGMEVTPSGTW | N_313-330_ | 93.19% |
| N-54 | RIGMEVTPSGTWLTYTGA | N_319-336_ | 92.58% |
| N-55 | TPSGTWLTYTGAIKLDDK | N_325-342_ | 95.50% |
| N-56 | LTYTGAIKLDDKDPNFKD | N_331-348_ | 92.94% |
| N-57 | IKLDDKDPNFKDQVILLN | N_337-354_ | 92.10% |
| N-58 | DPNFKDQVILLNKHIDAY | N_343-360_ | 92.99% |
| N-59 | QVILLNKHIDAYKTFPPT | N_349-366_ | 92.27% |
| N-60 | KHIDAYKTFPPTEPKKDK | N_355-372_ | 93.25% |
| N-61 | KTFPPTEPKKDKKKKADE | N_361-378_ | 93.93% |
| N-62 | EPKKDKKKKADETQALPQ | N_367-384_ | 93.40% |
| N-63 | KKKADETQALPQRQKKQQ | N_373-390_ | 94.49% |
| N-64 | TQALPQRQKKQQTVTLLP | N_379-396_ | 91.84% |
| N-65 | RQKKQQTVTLLPAADLDD | N_385-402_ | 92.53% |
| N-66 | TVTLLPAADLDDFSKQLQ | N_391-408_ | 96.22% |
| N-67 | AADLDDFSKQLQQSMSSA | N_397-414_ | 95.69% |
| N-68 | FSKQLQQSMSSADSTQA | N_403-419_ | 96.63% |

**Supplementary Table 2: SARS‐CoV‐2** **peptide libraries used in the study**

| SARS-CoV-2：nucleocapsid | | | | |
| --- | --- | --- | --- | --- |
| Peptide Libraries No | Peptide Sequence | Sequence No | Peptides Involved | Peptide Numbers |
| Pool 1 | msdngpqnqrnapritfggpsdstgsnqngersgarskqrrpqglpnntaswftaltqhgkedlkf | N_1-66_ | N-1~N-9 | 9 |
| Pool 2 | altqhgkedlkfprgqgvpintnsspddqigyyrratrrirggdgkmkdlsprwyfyylg | N_55-114_ | N-10~N-17 | 8 |
| Pool 3 | dlsprwyfyylgtgpeaglpygankdgiiwvategalntpkdhigtrnpannaaivlq lp | N_103-162_ | N-18~N-25 | 8 |
| Pool 4 | pannaaivlqlpqgttlpkgfyaegsrggsqassrsssrsrnssrnstpgssrgtsparm | N_151-210_ | N-26~N-33 | 8 |
| Pool 5 | pgssrgtsparmagnggdaalalllldrlnqleskmsgkgqqqqgqtvtkksaaeaskkp | N_199-258_ | N-34~N-41 | 8 |
| Pool 6 | tkksaaeaskkprqkrtatkaynvtqafgrrgpeqtqgnfgdqelirqgtdykhwpqiaqfapsas | N_247-312_ | N-42~N-50 | 9 |
| Pool 7 | wpqiaqfapsasaffgmsrigmevtpsgtwltytgaiklddkdpnfkdqvillnkhidayktfppt | N_301-366_ | N-51~N-59 | 9 |
| Pool 8 | khidayktfpptepkkdkkkkadetqalpqrqkkqqtvtllpaadlddfskqlqqsmssadstqa | N_355-419_ | N-60~N-68 | 9 |

**Supplementary Table 3: ELISA data related to the peptide libraries in the epitope mapping**

| **IgG** | **Patient No** | **OD450nm** | | | | | | | | | |
| --- | --- | --- | --- | --- | --- | --- | --- | --- | --- | --- | --- |
|  |  | **OVA**  **_323-339_** | **N** | **Pool1** | **Pool2** | **Pool3** | **Pool4** | **Pool5** | **Pool6** | **Pool7** | **Pool8** |
|  | **G01** | 0.287 | 1.562 | 0.214 | 0.277 | 0.249 | 0.336 | 0.390 | 0.111 | 0.254 | 0.940 |
|  | **G02** | 0.148 | 2.025 | 0.169 | 0.127 | 0.164 | 0.161 | 0.126 | 0.134 | 0.186 | 0.444 |
|  | **G03** | 0.203 | 1.727 | 0.191 | 0.092 | 0.167 | 0.258 | 0.276 | 0.296 | 0.251 | 0.471 |
|  | **G04** | 0.128 | 2.850 | 0.228 | 0.248 | 0.200 | 0.182 | 0.572 | 0.487 | 0.598 | 1.022 |
|  | **G05** | 0.231 | 3.033 | 0.248 | 0.616 | 0.183 | 0.239 | 0.270 | 0.378 | 0.218 | 0.889 |
|  | **G06** | 0.128 | 1.129 | 0.127 | 0.124 | 0.134 | 0.142 | 0.138 | 0.139 | 0.133 | 0.265 |
|  | **G07** | 0.183 | 0.482 | 0.165 | 0.163 | 0.139 | 0.160 | 0.155 | 0.147 | 0.182 | 0.272 |
|  | **G08** | 0.320 | 2.120 | 0.452 | 0.334 | 0.277 | 0.518 | 0.382 | 0.348 | 0.391 | 0.559 |
|  | **G09** | 0.279 | 3.528 | 0.266 | 0.356 | 0.283 | 1.115 | 0.301 | 0.348 | 0.221 | 2.062 |
|  | **G10** | 0.185 | 2.894 | 0.054 | 0.200 | 0.147 | 0.263 | 0.124 | 0.092 | 0.331 | 0.216 |
|  | **G11** | 0.150 | 1.867 | 0.200 | 0.200 | 0.165 | 0.253 | 0.148 | 0.227 | 0.197 | 0.213 |
|  | **G12** | 0.156 | 3.465 | 0.070 | 0.143 | 0.391 | 0.196 | 0.269 | 0.310 | 0.105 | 0.725 |
|  | **G13** | 0.191 | 1.852 | 0.181 | 0.208 | 0.098 | 0.347 | 0.211 | 0.385 | 0.292 | 0.395 |
| **IgM** | **M01** | 0.198 | 0.374 | 0.183 | 0.188 | 0.116 | 0.200 | 0.141 | 0.448 | 0.164 | 0.432 |
|  | **M02** | 0.248 | 0.501 | 0.238 | 0.254 | 0.219 | 0.310 | 0.300 | 0.392 | 0.299 | 0.682 |
|  | **M03** | 0.217 | 0.563 | 0.153 | 0.158 | 0.167 | 0.176 | 0.273 | 0.347 | 0.235 | 0.348 |
|  | **M04** | 0.223 | 0.422 | 0.411 | 0.360 | 0.341 | 0.524 | 0.439 | 0.593 | 0.425 | 0.961 |
|  | **M05** | 0.251 | 0.522 | 0.437 | 0.317 | 0.328 | 0.395 | 0.300 | 0.415 | 0.283 | 0.466 |
|  | **M06** | 0.247 | 0.828 | 0.260 | 0.208 | 0.279 | 0.307 | 0.312 | 0.399 | 0.260 | 0.489 |
|  | **M07** | 0.252 | 0.927 | 0.410 | 0.331 | 0.322 | 0.327 | 0.348 | 0.409 | 0.401 | 0.654 |
|  | **M08** | 0.278 | 0.640 | 0.301 | 0.221 | 0.270 | 0.433 | 0.254 | 0.347 | 0.247 | 0.525 |
|  | **M09** | 0.208 | 0.502 | 0.285 | 0.200 | 0.204 | 0.246 | 0.258 | 0.272 | 0.227 | 0.440 |
|  | **M10** | 0.243 | 0.700 | 0.256 | 0.177 | 0.176 | 0.405 | 0.220 | 0.446 | 0.203 | 0.364 |
|  | **M11** | 0.252 | 0.438 | 0.251 | 0.225 | 0.237 | 0.306 | 0.381 | 0.338 | 0.258 | 0.672 |
|  | **M12** | 0.252 | 0.503 | 0.156 | 0.204 | 0.155 | 0.244 | 0.256 | 0.257 | 0.139 | 0.93 |
|  | **M13** | 0.290 | 0.479 | 0.371 | 0.316 | 0.262 | 0.436 | 0.385 | 0.380 | 0.302 | 0.605 |

**Supplementary Table 4: ELISA data related to the individual peptide in the epitope mapping**

| **IgG** | **Patient No** | **OD450nm** | | | | | | | | | | |
| --- | --- | --- | --- | --- | --- | --- | --- | --- | --- | --- | --- | --- |
|  |  | **OVA**  **_323-339_** | **N** | **N****_355-372_** | **N_361-378_** | **N_367-384_** | **N_373-390_** | **N_379-396_** | **N_385-402_** | **N_391-408_** | **N_397-414_** | **N_403-419_** |
|  | **G01** | 0.284 | 1.744 | 0.180 | 0.307 | 0.232 | 0.457 | 0.276 | 0.167 | 1.024 | 0.264 | 0.333 |
|  | **G02** | 0.147 | 2.037 | 0.102 | 0.192 | 0.161 | 0.592 | 0.291 | 0.217 | 0.627 | 0.314 | 0.304 |
|  | **G03** | 0.202 | 1.835 | 0.152 | 0.168 | 0.177 | 0.259 | 0.379 | 0.304 | 0.848 | 0.349 | 0.288 |
|  | **G04** | 0.168 | 1.303 | 0.190 | 0.175 | 0.298 | 0.375 | 0.325 | 0.321 | 0.631 | 0.131 | 0.242 |
|  | **G05** | 0.182 | 3.669 | 0.152 | 0.145 | 0.137 | 0.154 | 0.318 | 0.265 | 0.369 | 0.181 | 0.111 |
|  | **G06** | 0.267 | 1.491 | 0.309 | 0.209 | 0.253 | 0.298 | 1.205 | 0.314 | 0.403 | 0.278 | 0.262 |
|  | **G07** | 0.221 | 0.675 | 0.168 | 0.225 | 0.282 | 0.440 | 1.189 | 0.334 | 0.402 | 0.339 | 0.398 |
|  | **G08** | 0.294 | 1.963 | 0.293 | 0.231 | 0.353 | 0.391 | 0.291 | 0.314 | 0.545 | 0.327 | 0.318 |
|  | **G09** | 0.161 | 3.818 | 1.964 | 0.278 | 0.173 | 0.130 | 0.178 | 2.181 | 0.557 | 0.264 | 0.149 |
|  | **G12** | 0.145 | 1.966 | 0.081 | 0.172 | 0.143 | 0.196 | 0.751 | 0.304 | 0.272 | 0.258 | 0.194 |
|  | **G13** | 0.188 | 1.197 | 0.242 | 0.266 | 0.227 | 0.456 | 0.874 | 0.106 | 0.352 | 0.343 | 0.304 |
| **IgM** | **M02** | 0.298 | 0.588 | 0.472 | 0.332 | 0.562 | 0.577 | 0.523 | 0.572 | 0.755 | 0.453 | 0.500 |
|  | **M03** | 0.255 | 0.651 | 0.340 | 0.229 | 0.384 | 0.294 | 0.376 | 0.702 | 0.562 | 0.307 | 0.340 |
|  | **M04** | 0.198 | 0.466 | 0.181 | 0.168 | 0.178 | 0.199 | 0.618 | 0.537 | 0.853 | 0.359 | 0.457 |
|  | **M05** | 0.213 | 0.452 | 0.388 | 0.191 | 0.348 | 0.376 | 0.445 | 0.583 | 0.501 | 0.289 | 0.337 |
|  | **M06** | 0.248 | 0.664 | 0.324 | 0.147 | 0.410 | 0.402 | 0.427 | 0.355 | 0.636 | 0.310 | 0.316 |
|  | **M07** | 0.281 | 0.885 | 0.329 | 0.349 | 0.355 | 0.430 | 0.359 | 0.357 | 0.537 | 0.334 | 0.421 |
|  | **M08** | 0.246 | 0.463 | 0.437 | 0.155 | 0.307 | 0.487 | 0.211 | 0.308 | 0.375 | 0.153 | 0.192 |
|  | **M09** | 0.226 | 0.542 | 0.437 | 0.228 | 0.243 | 0.219 | 0.457 | 0.435 | 0.368 | 0.291 | 0.391 |
|  | **M11** | 0.253 | 0.439 | 0.426 | 0.342 | 0.509 | 0.477 | 0.386 | 0.532 | 0.729 | 0.255 | 0.342 |
|  | **M12** | 0.199 | 0.408 | 1.264 | 0.173 | 0.798 | 0.600 | 0.312 | 0.361 | 0.982 | 0.213 | 0.201 |
|  | **M13** | 0.245 | 0.387 | 0.391 | 0.346 | 0.384 | 0.464 | 0.532 | 0.774 | 0.786 | 0.409 | 0.370 |
